# Number of natural teeth, denture use and mortality in Chinese elderly: a population-based prospective cohort study

**DOI:** 10.1186/s12903-020-01084-9

**Published:** 2020-04-10

**Authors:** Jin-Qiu Yuan, Yue-Bin Lv, Virginia Byers Kraus, Xiang Gao, Zhao-Xue Yin, Hua-Shuai Chen, Jie-Si Luo, Yi Zeng, Chen Mao, Xiao-Ming Shi

**Affiliations:** 1 Clinical Research Center, The Seventh Affiliated Hospital; Scientific Research Center, The Seventh Affiliated Hospital, Sun Yat-sen University, Shenzhen, 518107 Guangdong China; 2 National Institute of Environmental Health, Chinese Center for Disease Control and Prevention, #7 Panjiayuan Nanli, Chaoyang, Beijing, 100021 China; 3 Duke Molecular Physiology Institute and Division of Rheumatology, Department of Medicine, Duke University School of Medicine, Durham, North Carolina USA; 4 Nutritional Epidemiology Lab, Pennsylvania State University, Philadelphia, PA USA; 5 Division of Non-Communicable Disease Control and Community Health, Chinese Center for Disease Control and Prevention, Beijing, China; 6 Center for the Study of Aging and Human Development and the Geriatric Division of School of Medicine, Duke University, Durham, North Carolina USA; 7 Center for Study of Healthy Aging and Development Studies, Peking University, Beijing, China; 8 Division of Epidemiology, School of Public Health, Southern Medical University, Guangzhou, 510515 Guangdong China

**Keywords:** Cohort study, Denture, Mortality, Tooth loss

## Abstract

**Background:**

The associations between the number of natural teeth/denture use and all-cause mortality remain unclear due to lake of investigation for the potential interaction between tooth loss and denture use and for the potential changes in these exposures over time in older adults. We undertake this study to evaluate the associations of the number of natural teeth and/or denture use with mortality in Chinese elderly.

**Methods:**

This is a prospective cohort study of 36,283 older adults (median age: 90). The number of natural teeth and denture use were collected with structured questionnaire. We evaluated hazard ratios (HRs) and confidence intervals (CIs) using a Cox proportional hazards model adjusting for demographic factors, education, income, lifestyle factors, and comorbidities.

**Results:**

We documented 25,857 deaths during 145,947 person-years of observation. Compared to those with 20+ teeth, tooth loss was associated with a gradual increase in mortality, with an adjusted HR of 1.14 (95% CI, 1.06 to 1.23) for those with 10–19 teeth, 1.23 (95% CI, 1.15 to 1.31) for those with 1–9 teeth, and 1.35 (95% CI, 1.26 to 1.44) for those without natural teeth. Denture use was associated with lower risk of mortality (adjusted HR 0.81; 95% CI, 0.77 to 0.84). Subgroup analyses indicated that the benefit of denture use was greater in men than in women (*P* = 0.02) and tended to decrease with age (*P* < 0.001). The effects of denture use did not differ among various degrees of tooth loss (*P* = 0.17).

**Conclusions:**

Tooth loss was associated with an increased risk of mortality in older adults. Denture use provided a protective effect against death for all degrees of tooth loss however, this effect appeared to be modified by sex and age.

## Background

Teeth play an important physiological role in human, which affect chewing, swallowing, speaking, facial aesthetics, and social interactions [1]. Tooth loss represents a major health problem, particularly for elderly [1]. The prevalence of edentulism increases with age and varies among countries [2, 3]. The global age-standardized prevalence of edentulism in 2010 was 2.4% [2]. Although a decline in tooth loss and edentulism has been observed in the past decade [2], the World Health Organization goal of retaining at least 20 teeth at the age of 80 years has not yet been met in most countries [4].

A number of prospective studies have found an association between tooth loss and all-cause mortality, cardiovascular and cancer mortality [5–11]. Wearing dentures may reduce the mortality in older adults [7, 12, 13]. Tooth loss and denture use may affect mortality through inflammation, nutrition, masticatory function, facial appearance, and social engagement [14–18]. Despite a fair amount of previous research, many questions remain unclear. First, the shape of the association between teeth number and mortality, and the minimum teeth number that have no additional mortality risk were still unclear. Second, the associations with potential interactions, such as age and sex, were controversial in previous studies [19, 20]. Third, tooth loss and denture use are closely related, but the potential interaction between tooth loss and denture use, in relation to mortality, have not been comprehensively evaluated. Forth, many important confounders, like economic status, were not adjusted in most previous studies [5–13]. Fifth, previous studies evaluating denture use and mortality showed inconsistent results [13, 21]. Lastly, tooth loss and the denture use may change over time, but no previous studies have considered the variability over time in evaluating the effect. We carried out this study was to prospectively evaluate the associations of the number of natural teeth and/or denture use with mortality using the Chinese Longitudinal Healthy Longevity Survey (CLHLS) datasets.

## Methods

### Study design and participants

CLHLS is an ongoing longitudinal interview survey of over 40,000 old adults in 22 of 31 Chinese provinces. The included participants represent 985 million persons, about 85% of the national population [22]. The investigation began in 1998 and follow-up surveys were undertaken in 2000, 2002, 2005, 2008, 2011, and 2014, with approximately a 90% response rate in each wave. To maintain a large enough sample size, the CLHLS replaced the deceased respondents with new participants in the follow-up waves. To avoid the problem of small sub-sample sizes at the more advanced ages, the CLHLS interviewed nearly all centenarians [22]. Details of the CLHLS have been described elsewhere [22]. The study was approved by the Ethics Committee of Peking University and Duke University. Informed consents were obtained from all participants during the face-to-face interview.

**Fig. 1:**
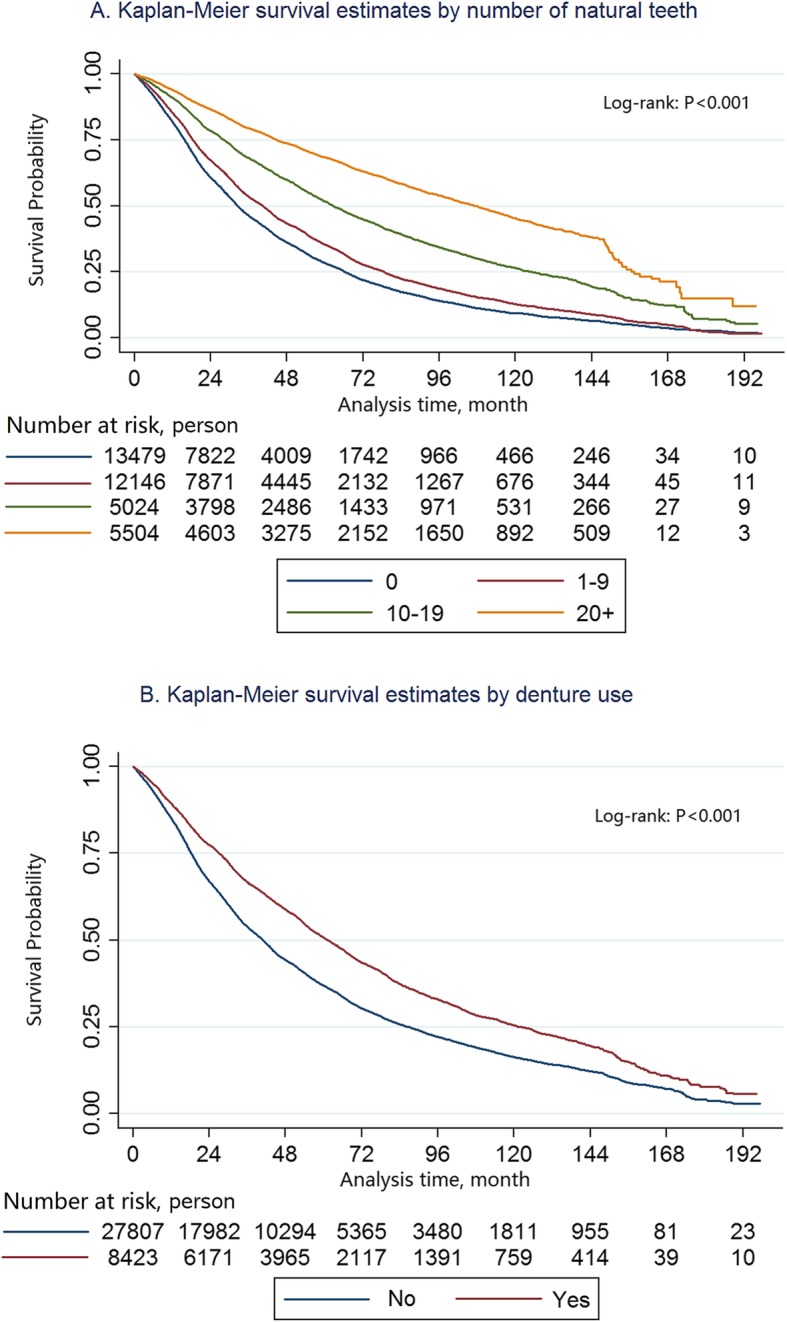
Kaplan–Meier plot showing the mortality by the number of natural teeth and denture use

Our inclusion criteria for participants were: aged 65 years or above; had data on the number of natural teeth and/or denture use; and data about the death time was available for at least one follow-up survey. We included all participants from the 1998 survey as all participants were newly recruited. For the follow-up surveys, the participants included the survivals from the previous waves and the new recruits. Only the new recruits needed to be included as the survivals from the previous waves had already been included. This strategy enabled us to analyze based on the largest sample size. In the end, we included 36,283 participants from the CLHLS, with 36,153 participants for the analysis of number of natural teeth and 36,230 for denture use (Figure 1 in the Additional file). There was a small difference in the participant numbers for the two analyses because some participants only reported one of the two exposure data.

### Assessment of the exposures

Self-reported number of natural teeth and the use of dentures were collected with the following questions: 1) How many natural teeth (teeth that are naturally grown) do you still have? 2) Do you have false teeth? (False teeth referred to any type of non-natural teeth, including partial or complete, removable or implant-retained fixed dentures). Data were repeatedly collected in each wave. We grouped the number of remaining teeth into four categories (0, 1–9, 10–19, and ≥ 20). We evaluated the combined effects by grouping the participants into 8 categories according to teeth number and denture use. Though the education level of included participants were low and one third of the subjects were cognitively impaired, the validity of self-reported number of natural teeth and denture use may not be influenced as the interviewers could help older adults to confirm their responses to these questions.

### Assessment of deaths

We ascertained the survival status through a face-to-face interview with a close family member for those interviewees who had died before the next wave [22]. The survival time was defined as the period from the date of the baseline visit to the date of death. The data for the participants who survived until the 2014 survey were censored at the time of the 2014 survey, and those who were lost to follow-up were censored at the time of the last survey.

### Assessment of covariates

We selected covariates that may confound the relationship based on a review of literature [23, 24]. The selected covariates should have an impact on the overall mortality and been collected in the CLHLS. For example, we included fresh fruit intake and vegetable consumption because they may influenced by teeth healthy and have an impact on mortality [25]. Because the sample size of this study is large and statistical power is strong, we included as many covariates as possible to minimize the potential confounding effect. We obtained covariate information from the structured questionnaire [26]. The covariates for our analyses included sociodemographic characteristics, routine physical checkup (weigh, height, and blood pressure), lifestyle behaviors (smoking, alcohol drinking, physical activity, fresh fruit intake, and vegetable consumption), self-reported medical history, activities of daily living (ADL), cognitive function (evaluated by the Mini-Mental State Examination, MMSE) [27], and depressive symptoms.

### Statistical analysis

We evaluated all-cause mortality according to the number of natural teeth and denture use with Kaplan-Meier survival plots. To explore the shape of the relationship, we used addictive Cox regression, taking number of natural teeth as a smoothed term. Penalized splines were used for smoothing. The degrees of freedom were determined according to Akaike Information Criterion and residual deviance. For better interpretation of effect, we also evaluated the hazard ratios (HRs) and 95% confidence intervals (CIs) with time-dependent Cox regression. We divided the subject’s time of observation into several intervals based on the time of follow-up surveys and date of death. The exposure values at the beginning of each interval were used for this interval.

Multivariable models were applied to adjust for confounding factors. We adjusted for age at baseline, sex, and residence (urban or rural, defined according to the official registration record for Chinese citizen) in the basic analysis model. Additionally, we adjusted for education, living arrangement, sufficient income for daily needs, smoking, alcohol drinking, frequent vegetable consumption, frequent fruit consumption, frequent physical activity, ADL, cognitive impairment (defined as MMSE < 24), body mass index, hypertension, self-reported diabetes mellitus, self-reported heart disease, self-reported cerebrovascular disease, self-reported respiratory disease in the fully-adjusted model. We coded the participants with missing covariate data to the reference group or median group when the missing rate was low (< 5%). When the rate of missing data was ≥5%, a separate missing response category was created. Based on the HRs and mortality rate, we calculated the age- and sex- specific number needed to treat (NNT) [28], which is conceptually easier to understand.

We conducted subgroup analyses by age, sex, residence, years of education, BMI, smoking status, and drinking status, in order to investigate potential effect-modifying effect. We also evaluated the association of teeth number with all-cause mortality by denture use and vice versa.

We conducted a number of sensitivity analyses: 1) additionally adjusting for depressive symptom, current marital status, time of recruitment; 2) using the baseline number of natural teeth and denture use as covariates; 3) excluding patients with a history of diabetes mellitus, heart disease, cerebrovascular disease, or respiratory diseases; 4) excluding participants with an observation time of < 3 years, > 12 years, or without tooth loss; 5) additionally adjusting for living location; and 6) considering the participants with unknown survival status censored at the median of follow-up (3 years) as previous study [29]; 6) excluding the participants who reported more number of natural teeth than the teeth number in the previous wave of survey. Analyses were completed using Stata version 12.0 (StataCorp LP, College Station, TX, USA) and R software version 3.4.1 (R Development Core Team, 2017).

## Results

### Baseline characteristics

Table 1 presents the baseline characteristics. The median age of participants was 90 years (interquartile range [IQR] 81 to 99) and 41.1% were men. Most of the participants (98.1%) had lost one or more teeth and 37.3% had lost all teeth. The overall denture use rate was 23.6% and increased with tooth loss.

**Table 1:** Characteristics of participants by the number of natural teeth and denture use

	Number of natural teeth (n = 36,153)					Denture use (*n* = 36,230)		
	20+	10–19	1–9	0	*P*-value^a^	Yes	No	*P*-value^a^
No. of participants	5504	5024	12,146	13,479		8423	27,807	
Median (IQR) age, years	74(67–84)	84(77–91)	91(84–100)	95(87–100)	< 0.001	86(79–94)	91(82–100)	< 0.001
Male, n(%)	3126(56.8)	2489(49.5)	4965(40.9)	4251(31.5)	< 0.001	4053(48.1)	10,840(39.0)	< 0.001
Residence, n(%)								
Urban	2349(42.7)	2054(40.9)	4444(36.6)	5368(39.8)	< 0.001	4361(51.8)	9919(35.7)	< 0.001
Rural	3155(57.3)	2970(59.1)	7702(63.4)	8111(60.2)		4062(48.2)	17,888(64.3)	
Education time, years								
0	2474(45.1)	2913(58.2)	8447(70.0)	9986(74.5)	< 0.001	4451(53.1)	19,374(70.1)	< 0.001
> =1	3009(54.9)	2090(41.8)	3624(30.0)	3414(25.5)		3935(46.9)	8269(29.9)	
Living arrangement								
Living alone	4870(88.6)	4260(84.8)	10,473(86.3)	11,927(88.5)	< 0.001	7335(87.1)	24,268(87.3)	0.60
With others	628(11.4)	762(15.2)	1668(13.7)	1546(11.5)		1086(12.9)	3523(12.7)	
Sufficient income for daily needs								
Yes, n(%)	3979(80.8)	3149(77.4)	6528(74.7)	7882(77.6)	< 0.001	5608(81.9)	15,974(75.7)	< 0.001
No, n(%)	948(19.2)	918(22.6)	2209(25.3)	2280(22.4)		1238(18.1)	5130(24.3)	
Median (IQR) BMI, kg/m^2^	20.5(18.2–23.6)	19.5(17.2–22.4)	18.5(16.3–21.1)	18.4(16.2–21.0)	< 0.001	19.8(17.7–22.7)	18.6(16.5–21.3)	< 0.001
Smoking, n(%)								
Non-smoker	3285(59.8)	3232(64.4)	8311(68.5)	9663(71.8)	< 0.001	5285(62.8)	19,240(69.3)	< 0.001
Current smoker	1406(25.6)	1025(20.4)	2180(18.0)	1998(14.8)		1692(20.1)	4938(17.8)	
Former smoker	804(14.6)	764(15.2)	1642(13.5)	1803(13.4)		1440(17.1)	3594(12.9)	
Alcohol consumption, n(%)								
Non-drinker	3455(62.9)	3383(67.4)	8264(68.1)	9806(72.9)	< 0.001	5770(68.6)	19,191(69.1)	0.272
Current drinker	1459(26.6)	1080(21.5)	2575(21.2)	2413(17.9)		1745(20.7)	5790(20.8)	
Former drinker	581(10.6)	558(11.1)	1290(10.6)	1241(9.2)		896(10.7)	2789(10.0)	
Frequent vegetable intake, n(%)	1859(33.8)	1829(36.4)	4765(39.2)	5546(41.2)	< 0.001	3030(36.0)	10,989(39.5)	< 0.001
Frequent fruit intake, n(%)	811(14.8)	563(11.2)	1262(10.4)	1841(13.7)	< 0.001	1679(19.9)	2821(10.2)	< 0.001
Frequent physical activity, n(%)	2242(40.8)	2033(40.5)	4899(40.4)	4889(36.3)	< 0.001	3713(44.1)	10,393(37.4)	< 0.001
Impaired cognitive function, n(%)	724(13.7)	1183(25.3)	4633(42.6)	5740(51.1)	< 0.001	1912(24.9)	10,387(42.5)	< 0.001
Restricted ADL, n(%)	605(11.0)	869(17.3)	3483(28.8)	5494(40.9)	< 0.001	2068(24.6)	8401(30.3)	< 0.001
Hypertension, n(%)	2548(47.9)	2133(43.9)	5051(43.3)	5598(43.9)	< 0.001	3457(42.7)	11,900(44.8)	0.001
Diabetes, n(%)	163(1.2)	153(1.3)	110(2.2)	155(2.8)	< 0.001	212(2.5)	371(1.3)	< 0.001
Heart disease, n(%)	982(7.3)	788(6.5)	417(8.3)	495(9.1)	< 0.001	935(11.2)	1758(6.4)	< 0.001
Cerebrovascular disease, n(%)	565(4.2)	478(4.0)	230(4.6)	304(5.6)	< 0.001	494(5.9)	1082(3.9)	< 0.001
Respiratory disease ^a^, n(%)	1540(11.5)	1364(11.3)	584(11.7)	587(10.7)	0.05	1078(12.9)	3009(10.9)	< 0.001
Denture use, n(%)								
Yes	652(11.9)	856(17.1)	1952(16.1)	4958(36.8)	< 0.001	NA	NA	NA
No	4845(88.1)	4161(82.9)	10,170(83.9)	8506(63.2)		NA	NA	
Number of natural teeth, n(%)								
20+	NA	NA	NA	NA	NA	652(7.7)	4845(17.5)	< 0.001
10–19	NA	NA	NA	NA		856(10.2)	4161(15.0)	
1–9	NA	NA	NA	NA		1952(23.2)	10,170(36.7)	
0	NA	NA	NA	NA		4958(58.9)	8506(30.7)	

*IQR* Interquartile range, *ADL* Activity of daily living, *BMI* Body mass index, *NA* Not available

The differences were tested by Kruskal-Wallis test or χ^2^ test

^a^ Including bronchitis, emphysema, asthma, pneumonia

### Number of natural teeth and all-cause mortality

The median follow-up time was 3 years (maximum: 16.5 years, IQR 1.6 to 5.7 years). During a total of 145,947 person-years of observation, we documented 25,857 deaths (71.3%). Figure 1**-A** suggested that the mortality among various teeth groups were significantly different (log-rank test: *P* < 0.001).

**Fig. 2:**
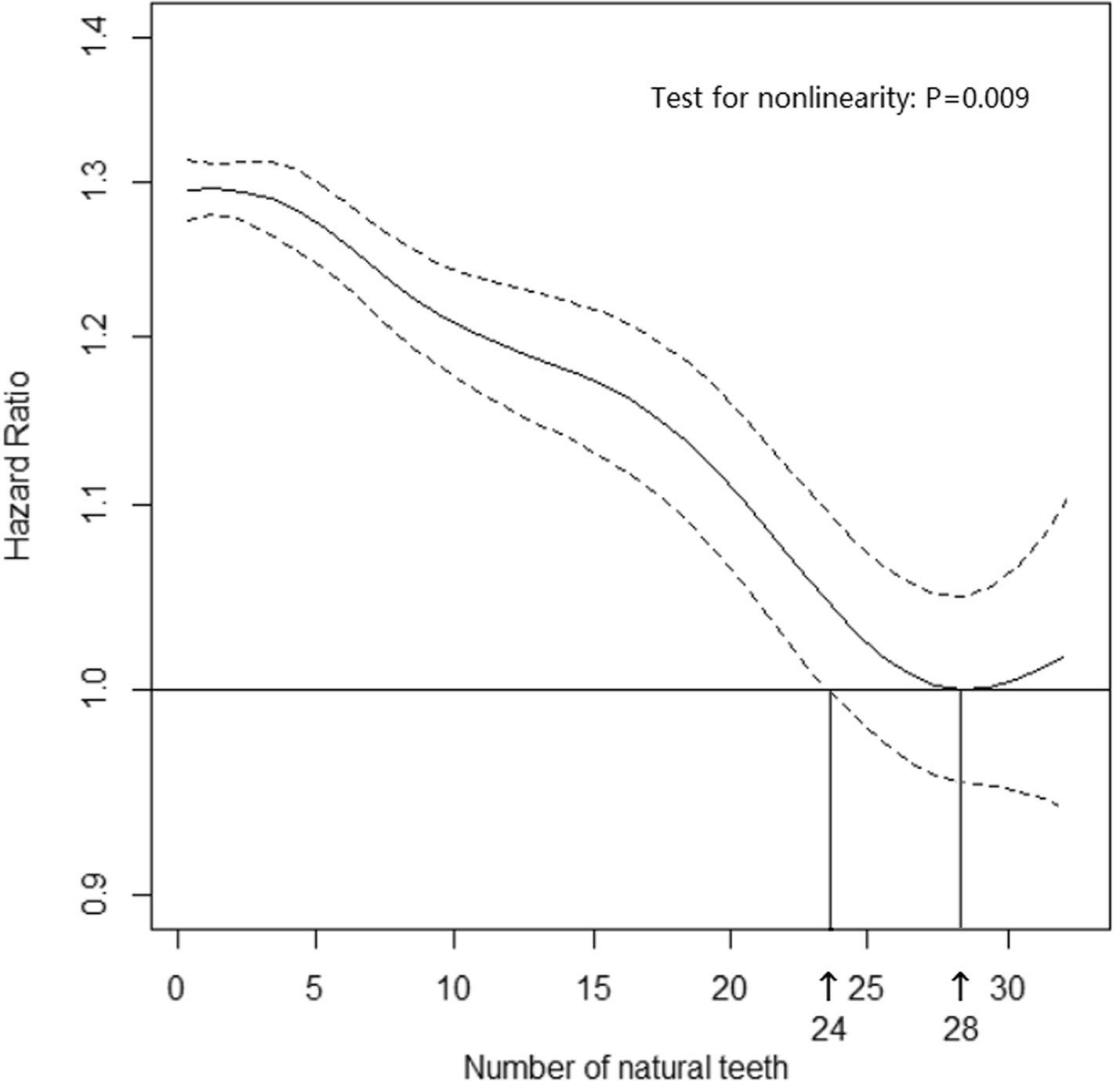
Association between the number of natural teeth and mortality. The results were based on addictive cox regression taking number of natural teeth as a smoothing term in the model. The inflection point with lowest hazard (28 teeth) was considered the reference. The addictive cox regression model has adjusted for age, sex, residence, denture use, education, sufficient income for daily needs, living arrangement, BMI, smoking, alcohol drinking, frequent vegetable consumption, frequent fruit consumption, impaired activity of daily living, cognitive impairment, hypertension, self-reported history of diabetes mellitus, self-reported history of heart disease, self-reported history of cerebrovascular disease, and self-reported history of respiratory diseases. The minimum number of natural teeth showing no significantly increased risk was 25 (HR for 24 teeth: 1.05, 95%CI 1.00 to1.10, P = 0.05; HR for 25 teeth: 1.03, 95%CI 0.99 to 1.08, P = 0.18)

Figure 2 presented the nonlinear association between the number of natural teeth and mortality. We considered the participants with 28 teeth as reference as they had the lowest risk. Twenty-eight is also the number of usable teeth in human. The association was hockey stick-shaped (Test for nonlinear: *P* = 0.009). The HRs first decreased with the number of natural teeth (approximately between 0 to 25 teeth) and then kept stable (approximately between 26 to 32 teeth). The minimum number of natural teeth showing no significantly increased mortality was 25 teeth (HR for 25 teeth: 1.03, 95% CI 0.99 to 1.08, *P* = 0.18; HR for 24 teeth: 1.05, 95% CI 1.00 to 1.10, *P* = 0.05).

Table 2 presents the HRs categorized by teeth number. Tooth loss was associated with a gradual increase in the risk of mortality (P-trend < 0.001), with an adjusted HR of 1.14 (95% CI, 1.06 to 1.23) for those with 10–19 teeth, 1.23 (95% CI, 1.15 to 1.31) for those with 1–9 teeth, and 1.35 (95% CI, 1.26 to 1.44) for those lacking natural teeth, as compared to those with 20+ teeth.

**Table 2:** Associations of the number of natural teeth or denture use with mortality

	Hazard Ratio[95% Confidence Interval]		
	Unadjusted model	Basic model ^a^	Fully adjusted model ^b^
**Number of natural teeth**			
No. of participants	36,153	36,100	20,816
No. of deaths	25,737	25,713	12,757
*20+*	1.00	1.00	1.00
*10–19*	1.59[1.51, 1.69]	1.13[1.07, 1.20]	1.14[1.06, 1.23]
*1–9*	2.60[2.48, 2.72]	1.29[1.23, 1.36]	1.23[1.15, 1.31]
*0*	3.18[3.03, 3.33]	1.46[1.38, 1.53]	1.35[1.26, 1.44]
P-trend	*< 0.001*	*< 0.001*	*< 0.001*
**Denture use**			
No. of participants	36,230	36,100	20,816
No. of deaths	25,824	25,713	12,757
*No*	1.00	1.00	1.00
*Yes*	0.66[0.64, 0.68]	0.76[0.74, 0.79]	0.81[0.77, 0.84]

*HR* Hazard ratio, *CI* Confidence interval;

^a^Basic model: adjusted for age (years), sex (men or women), and residence (urban or rural), teeth number (0, 1–9, 10–19, ≥20, for analysis of denture use), denture use (yes or no, for analysis of teeth number);

^b^Fully adjusted model: additionally adjusted for education (yes or no), sufficient income for daily needs (yes or no), living arrangement (living alone or with others), BMI (< 18.5, > = 18.5 and < 24, or > =24), smoking (current smoker, former smoker, or never smoker), alcohol drinking (current drinker, former drinker, or non- drinker), frequent vegetable consumption (yes or no), frequent fruit consumption (yes or no), impaired activity of daily living (yes or no), cognitive impairment(yes or not), hypertension (yes or not), self-reported history of diabetes mellitus (yes or no), self-reported history of heart disease (yes or no), self-reported history of cerebrovascular disease (yes or no), and self-reported history of respiratory diseases (yes or no)

### Denture use and all-cause mortality

Figure 1**-B** suggested that the mortality in denture users was lower than non-users (log-rank test *P* < 0.001). The individuals with dentures had lower risk of mortality as compared with those who did not (adjusted HR 0.81; 95% CI 0.77 to 0.84) (Table 2). Overall, 17.3 and 34.8 older adults need to wear dentures to prevent one death in 5 and 10 years, respectively. The NNTs increased with age and were larger in women than in men (Table 3).

**Table 3:** Age- and sex-specific number needed to treat to prevent one death in 5 and 10 years

	5-year mortality rate in non-denture users	10-year mortality rate in non-denture users	HR[95% CI] of denture users versus non-denture users	NNT[95% CI] to prevent one death in 5 years	NNT[95% CI] to prevent one death in 10 years
65–79 years & men	19.8%	44.7%	0.64[0.55, 0.74]	6.4[4.7, 9.7]	6.6[5.1, 9.6]
80–89 years & men	56.1%	85.7%	0.69[0.62, 0.78]	9.1[7.2, 12.9]	24.0[19.1, 33.4]
> = 90 years & men	82.4%	97.9%	0.83[0.75, 0.92]	36.7[24.6, 79.6]	287.3[193.7, 617.8]
65–79 years & women	16.7%	36.7%	0.66[0.56, 0.78]	7.3[5, 12.6]	6.8[5.0, 11.2]
80–89 years & women	48.9%	81.5%	0.79[0.70, 0.90]	12.8[8.5, 27.2]	28.3[19.4, 58.5]
> = 90 years & women	82.1%	97.7%	0.85[0.79, 0.93]	41.5[28.3, 82.9]	300[205.7, 595.5]

*HR* Hazard ratio, *CI* Confidence interval, *NNT* Number needed to treat

HRs were based on fully-adjusted cox regression models

### Combined effects of tooth loss and denture use

Among those who did not use dentures, the adjusted HRs as compared with the elderly without natural teeth were 0.91 (95%CI 0.87 to 0.96), 0.87 (95%CI 0.82 to 0.93), and 0.75 (95%CI 0.70 to 0.80) for those with 1–9, 10–19, and 20+ teeth, respectively (Table 1 in the Additional file). The estimated HRs tended to be larger for denture users than non-users in each category of teeth.

### Subgroup analyses

For the number of natural teeth, the comparisons of the 0 tooth group (*P* = 0.001) or 1–9 teeth group (*P* < 0.001) versus the reference group (20+ teeth) were different by age group; the risks tended be higher in the younger elderly (65–79 years) than in the older age group of 80+ (Table 2 in the Additional file*)*. Subgroup analyses for number of natural teeth by sex, residence status, BMI, smoking status, alcohol drinking, and denture use did not show any interaction effects.

Subgroup analyses for denture use showed interaction effects with age (*P* < 0.001) and sex (*P* = 0.02). The benefit of denture use tended to decrease with age, with a fully-adjusted HR of 0.65 for those aged 65–79 years, 0.73 for those aged 80–89 years, and 0.84 for those aged 90 and over. Denture use was associated with a greater reduction in mortality in men (HR 0.76; 95% CI 0.71 to 0.82) than in women (HR 0.84; 95% CI 0.79 to 0.89).

### Sensitivity analysis

Our sensitivity analyses, additionally adjusting for depressive symptom, marital status, time of recruitment, and living location yielded results largely similar to the primary results (Table 3 in the Additional file). The effect size by Cox regression analyses, evaluating the baseline number of natural teeth and denture use, was slightly smaller than the primary results, but the conclusion was unchanged. Sensitivity analyses by excluding participants with an observation time of < 3 years, > 12 years, without loss of teeth, with increased number of natural teeth between two waves of surveys, and considering the older adults with unknown survival status censored at the median of follow-up (3 years) showed no major difference.

## Discussion

This cohort study of 36,283 Chinese older adults suggested there was likely to be a hockey stick-shaped association between the number of natural teeth and all-cause mortality. The mortality risk decreased with increment of number of natural teeth, and those who had 24 teeth or less were associated with significantly increased risk of mortality. This finding suggested that 25 teeth is the minimum number of natural teeth to avoid extra risk of death. Denture use was associated with a decreased risk of all-cause mortality that tended to wane with age. The absolute benefit of wearing dentures was large and varied with age. In order to prevent one death in 5 years, approximately 6 to 7 older people aged 56–79 need to wear dentures, compared with 37 to 42 people for those aged 95 and over. The benefit of wearing dentures was similar among different degrees of tooth loss. The primary results were robust as shown in a series of sensitivity analyses.

Our findings are consistent with previous analyses in older populations [7–9, 12]. In a cohort study of 21,730 individuals, the number of natural teeth was inversely associated with mortality [7]. A retrospective cohort study of 55,651 old adults suggested that the HRs of all-cause mortality in the participants with no teeth, 1–9 teeth, and 10–19 teeth were 1.36, 1.24, and 1.19, respectively, which were similar to our estimates [8]. In the Golestan Cohort Study, wearing dentures reduced all-cause mortality by 10% [12]. The effect was smaller than our estimate (19%), possibly due to the difference in ethnicity and age.

Previous studies yielded mixed results about the interaction of age and sex on the association between teeth number and mortality. A cohort study suggested that number of natural teeth was inversely associated with all-cause mortality among the individuals aged 40–64 years but not among those aged 65–79 years [5]; However, an association was shown among adults aged 65 and older in another cohort study [7]. In a cohort study of 1282 subjects aged 80 years, tooth loss was a predictor of mortality in women but not in men [9]; However, in a 10-year cohort study of 118 subjects aged 80 years or over, an association between number of natural teeth and survival rate was shown in men but not in women [10]. A possible explanation is that the sample sizes in subgroups in these studies were too small. Our subgroup analyses by age did show between-group differences, but the association persisted in most subgroups.

Our subgroup analysis suggested that the protective effect of denture use decreased with age. The younger elders (65–79 years) had the highest risk of death due to tooth loss in our study; This more severely affected group may therefore have been more readily able to demonstrate a protective effect of wearing dentures. Our subgroup analysis by sex suggested that the effect of wearing dentures tended to be greater in men than in women. A possible explanation is that men tend to have a poorer nutritional status than women [30], while wearing dentures could effectively improve nutritional intake [31]; Therefore men are likely to have more benefits from denture use. Additionally, previous studies have shown that men are less active in social participation than women [32], while wearing dentures can encourage social participation [33]; Thus, it is expected that men will benefit more from denture placement. Only one prior study investigated denture use and mortality by sex. In this study, denture use was associated with lower mortality in women but not in men in individuals with less than 10 teeth; there were no major differences of mortality rates between subjects with 10 or more teeth with and without dentures or based on sex [34]. The sample size in this subgroup might be too small to test the difference.

Based on additive cox regression, we found that the risk of mortality decreased with increment of numbers of natural teeth. The risk was parallel to the severity of tooth loss and a statistically significant increased risk was observed at 24 or less teeth. This may be because more tooth loss could have a more severe influence on the masticatory function and nutritional status, which in turn, be linked to overall morality. The elderly with 24 or less teeth had ≥5% increased risk. For the elderly with 25 or more teeth, there was no sufficient evidence of increased risk, and if any, the risk would be less than 3%.

In addition to the burden of inflammation, another possible mechanism underlying the association of tooth loss and mortality could be nutrition. Fewer teeth are associated with impaired masticatory function and nutritional status [14, 15], which in turn, increases the risk of mortality [35, 36]. This was consistent with our analysis of baseline characteristics, which suggested that the individuals with fewer teeth had lower BMI. Denture may reduce mortality by improving masticatory function, bite force, and nutritional state [16]. Use of dentures may also benefit older adults through preventing foreign body asphyxiation, enhancing phonetics, improving facial appearance, and facilitating social engagement [17, 18].

The use of dentures in older adults could be influenced by the cost. Cost for dentures varies greatly depending on type of dentures, type of materials used, additional necessary equipment, and region. For example, the cost for one replacement tooth in China ranged from approximately 60 USD to 1500 USD [37]. In China, a variety of dentures are available for older adults, adapted to their income. The overall denture use rate, particularly in those with no or few remaining teeth, was relatively low in this study as compared with the older adults from Western Europe or Japan. This is because denture use rate is closely related to economic status, while China is a developing country and 60.6% of the included participants were from rural area with low income.

The strengths of this study included the large sample size, the prospective cohort study design, investigation of the optimal teeth number and the shape of association, and evaluation of the interaction between teeth number and denture use. In addition, we used a time-dependent Cox proportional model which could not only control the baseline number of natural teeth/denture use, but also control the change in these exposures during follow-up. Furthermore, our sensitivity analyses were robust, which could reduce potential bias from additional confounders (such as marital status), participants with extreme short or long survival, and different methods for handling censored data. Lastly, this study also found solid evidence that the protective effect of denture use was modified by age and sex.

Our study has several limitations. First, residual confounding by other unmeasured or unknown factors remains possible. Second, we cannot investigate the type of dentures, cause of death, time of tooth loss and denture use, dental symptoms, and dental care/utilization, as these data were not collected. Third, our study included a sample with very old age (median age: 90 years). The participants are older than the general elder population in China. The main study findings, including the evaluation for the optimal number of natural teeth, may not be applicable to other population. Fourth, the primary results may be influenced by the exclusion of 6642 participants whose survival status was unknown as they might have different characteristics. However, our subgroup analyses suggested that the primary effects were not modified by most baseline characteristics. The sensitivity analysis by considering the older adults with unknown survival status censored at the median of follow-up (3 years) suggested that the primary results were robust. Fifth, According to the 4th National Oral Health Survey, the mean remaining teeth was 22.5 ± 8.7 and denture use rate was 34.0% in the 65 to 74 age group in 2015–2016 in China (data for the elderly aged 75 and over was not collected) [38]. The mean remaining teeth (17.6 ± 10.6) and denture use rate (29.9%) in this age group were relatively lower in our study. This was because our data was collected much earlier than this national survey and there was a continuous improvement in oral health of Chinese elderly in the past decades. This may influence the generalizability of our conclusion. Sixth, there is a threat that some older adults whom died soon after recruitment may not be due to tooth loss or denture use. We undertook sensitivity analysis by excluding participants with an observation time of < 3 years and the result showed no major differences as compared to the primary results. Therefore, we did not include an induction period in the primary results as most previous studies. Last, the quality of evidence is low due to the observational study design.

## Conclusion

In conclusion, our results indicate that tooth loss was associated with an increased risk of all-cause mortality. The optimal number of natural teeth in older adults was ≥25. Wearing dentures might have a protective effect against death, and the effect tended to decrease with age and be greater in men than in women. Preventing tooth loss and using dentures would have substantial public health benefits in view of the high prevalence of tooth loss and low denture use rate in this rapidly growing population.

## Supplementary information

**Additional file 1: Figure S1.** Flowchart of participant enrollment

**Additional file 2: Table S1.** Combined effects of tooth loss and denture use on mortality

**Additional file 3: Table S2.** Subgroup analysis for the associations between the number of natural teeth or denture use with mortality

**Additional file 4: Table S3.** Sensitivity analysis for the association of the number of natural teeth or denture use with mortality

## Data Availability

This study was based on the datasets from the Chinese Longitudinal Healthy Longevity Survey (CLHLS) in longevity areas. The CLHLS data can be publicly obtained through the National Archive of Computerized Data on Aging (NACDA) (https://www.icpsr.umich.edu/icpsrweb/NACDA/series/487).

## Abbreviations

CLHLS: Chinese longitudinal healthy longevity survey
BMI: Body mass index
HR: Hazard ratios
CI: Confidence interval
ADL: Activities of daily living
MMSE: Mini-mental state examination
NNT: Number needed to treat
IQR: Interquartile range

## References

[1] Sischo L, Broder HL (2011). Oral health-related quality of life. What, why, how, and future implications. J Dent Res.

[2] Kassebaum NJ, Bernabe E, Dahiya M, Bhandari B, Murray CJ, Marcenes W (2014). Global burden of severe tooth loss: a systematic review and meta-analysis. J Dent Res.

[3] Peltzer K, Hewlett S, Yawson AE, Moynihan P, Preet R, Wu F (2014). Prevalence of loss of all teeth (Edentulism) and associated factors in older adults in China, Ghana, India, Mexico, Russia and South Africa. Int J Environ Res Public Health.

[4] Muller F, Naharro M, Carlsson GE (2007). What are the prevalence and incidence of tooth loss in the adult and elderly population in Europe?. Clin Oral Implants Res.

[5] Ando A, Tanno K, Ohsawa M, Onoda T, Sakata K, Tanaka F (2014). Associations of number of teeth with risks for all-cause mortality and cause-specific mortality in middle-aged and elderly men in the northern part of Japan: the Iwate-KENCO study. Community Dent Oral Epidemiol.

[6] Paganini-Hill A, White SC, Atchison KA (2011). Dental health behaviors, dentition, and mortality in the elderly: the leisure world cohort study. J Aging Res.

[7] Hayasaka K, Tomata Y, Aida J, Watanabe T, Kakizaki M, Tsuji I (2013). Tooth loss and mortality in elderly Japanese adults: effect of oral care. J Am Geriatr Soc.

[8] Hu HY, Lee YL, Lin SY, Chou YC, Chung D, Huang N (2015). Association between tooth loss, body mass index, and all-cause mortality among elderly patients in Taiwan. Medicine..

[9] Ansai T, Takata Y, Soh I, Awano S, Yoshida A, Sonoki K (2010). Relationship between tooth loss and mortality in 80-year-old Japanese community-dwelling subjects. BMC Public Health.

[10] Morita I, Nakagaki H, Kato K, Murakami T, Tsuboi S, Hayashizaki J (2006). Relationship between survival rates and numbers of natural teeth in an elderly Japanese population. Gerodontology..

[11] Koka S, Gupta A (2017). Association between missing tooth count and mortality: a systematic review. J Prosthodont Res.

[12] Vogtmann E, Etemadi A, Kamangar F, Islami F, Roshandel G, Poustchi H (2017). Oral health and mortality in the Golestan cohort study. Int J Epidemiol.

[13] Shimazaki Y, Soh I, Saito T, Yamashita Y, Koga T, Miyazaki H (2001). Influence of dentition status on physical disability, mental impairment, and mortality in institutionalized elderly people. J Dent Res.

[14] Nowjack-Raymer RE, Sheiham A (2007). Numbers of natural teeth, diet, and nutritional status in US adults. J Dent Res.

[15] Hung HC, Willett W, Ascherio A, Rosner BA, Rimm E, Joshipura KJ (2003). Tooth loss and dietary intake. J Am Dent Assoc.

[16] Boven GC, Raghoebar GM, Vissink A, Meijer HJ (2015). Improving masticatory performance, bite force, nutritional state and patient's satisfaction with implant overdentures: a systematic review of the literature. J Oral Rehabil.

[17] Kikutani T, Tamura F, Tohara T, Takahashi N, Yaegaki K (2012). Tooth loss as risk factor for foreign-body asphyxiation in nursing-home patients. Arch Gerontol Geriatr.

[18] Felton D, Cooper L, Duqum I, Minsley G, Guckes A, Haug S (2011). Evidence-based guidelines for the care and maintenance of complete dentures: a publication of the American College of Prosthodontists. J Prosthodont.

[19] Polzer I, Schwahn C, Volzke H, Mundt T, Biffar R (2012). The association of tooth loss with all-cause and circulatory mortality. Is there a benefit of replaced teeth? A systematic review and meta-analysis. Clin Oral Investig.

[20] Gerritsen AE, Allen PF, Witter DJ, Bronkhorst EM, Creugers NH (2010). Tooth loss and oral health-related quality of life: a systematic review and meta-analysis. Health Qual Life Outcomes.

[21] Janket SJ, Surakka M, Jones JA, Lam A, Schnell RA, Rose LM (2013). Removable dental prostheses and cardiovascular survival: a 15-year follow-up study. J Dent.

[22] Zeng Y (2012). Towards deeper research and better policy for healthy aging --using the unique data of Chinese longitudinal healthy longevity survey. China Economic J.

[23] Fried LP, Kronmal RA, Newman AB, Bild DE, Mittelmark MB, Polak JF (1998). Risk factors for 5-year mortality in older adults: the Cardiovascular Health Study. JAMA.

[24] Maia Fde O, Duarte YA, Lebrao ML, Santos JL (2006). Risk factors for mortality among elderly people. Rev Saude Publica.

[25] Wang X, Ouyang Y, Liu J, Zhu M, Zhao G, Bao W (2014). Fruit and vegetable consumption and mortality from all causes, cardiovascular disease, and cancer: systematic review and dose-response meta-analysis of prospective cohort studies. BMJ.

[26] Russell SL, Gordon S, Lukacs JR, Kaste LM (2013). Sex/Gender differences in tooth loss and edentulism: historical perspectives, biological factors, and sociologic reasons. Dent Clin N Am.

[27] Katzman R, Zhang MY, Ouang Ya Q, Wang ZY, Liu WT, Yu E (1988). A Chinese version of the mini-mental state examination; impact of illiteracy in a Shanghai dementia survey. J Clin Epidemiol.

[28] Altman DG, Andersen PK (1999). Calculating the number needed to treat for trials where the outcome is time to an event. BMJ.

[29] Lv YB, Gao X, Yin ZX, Chen HS, Luo JS, Brasher MS (2018). Revisiting the association of blood pressure with mortality in oldest old people in China: community based, longitudinal prospective study. BMJ.

[30] Boscatto EC, Duarte Mde F, Coqueiro Rda S, Barbosa AR (2013). Nutritional status in the oldest elderly and associated factors. Rev Assoc Med Bras (1992).

[31] Gupta A, Khandelwal R, Kapil U (2019). Interrelationship between dental health status and nutritional status among elderly subjects in India. J Family Med Prim Care.

[32] Tomioka K, Kurumatani N, Hosoi H (2017). Age and gender differences in the association between social participation and instrumental activities of daily living among community-dwelling elderly. BMC Geriatr.

[33] Dable RA, Nazirkar GS, Singh SB, Wasnik PB (2013). Assessment of Oral health related quality of life among completely edentulous patients in Western India by using GOHAI. J Clin Diagn Res.

[34] Fukai K, Takiguchi T, Ando Y, Aoyama H, Miyakawa Y, Ito G (2008). Mortality rates of community-residing adults with and without dentures. Geriatr Gerontol Int.

[35] de Groot LC, Verheijden MW, de Henauw S, Schroll M, van Staveren WA, Investigators S (2004). Lifestyle, nutritional status, health, and mortality in elderly people across Europe: a review of the longitudinal results of the SENECA study. J Gerontol A Biol Sci Med Sci.

[36] Soderstrom L, Rosenblad A, Thors Adolfsson E, Bergkvist L (2017). Malnutrition is associated with increased mortality in older adults regardless of the cause of death. Br J Nutr.

[37] Li Y (2012). Expensive dentures. China Qual Promotion.

[38] Guo J, Ban JH, Li G, Wang X, Feng XP, Tai BJ (2018). Status of tooth loss and denture restoration in Chinese adult population: findings from the 4th National Oral Health Survey. Chin J Dent Res.

