# Supplementary figures and images for: Number of natural teeth, denture use and mortality in Chinese elderly: a population-based prospective cohort study
Source: BMC Oral Health. 2020 Apr 10;20:100. doi: 10.1186/s12903-020-01084-9 (PMC7147045; doi:10.1186/s12903-020-01084-9)

**Figure S1.** Flowchart of participant enrollment

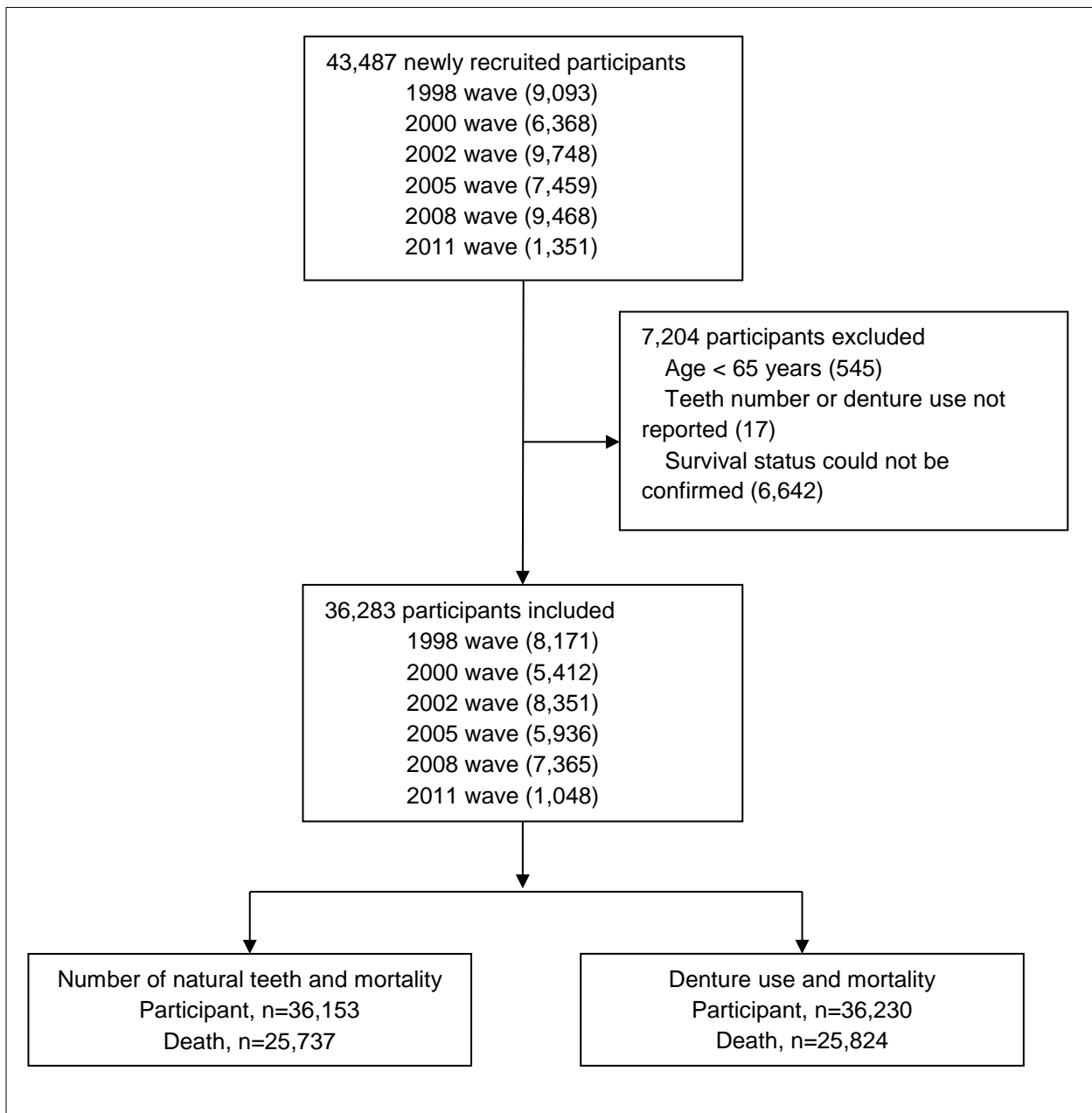

Supplement: Supplementary file 1 — Additional file 1: Figure S1. Flowchart of participant enrollment [file 12903_2020_1084_MOESM1_ESM.pdf]
