# Supplementary material for: Number of natural teeth, denture use and mortality in Chinese elderly: a population-based prospective cohort study
Source: BMC Oral Health. 2020 Apr 10;20:100. doi: 10.1186/s12903-020-01084-9 (PMC7147045; doi:10.1186/s12903-020-01084-9)
Supplement: Supplementary file 2 — Additional file 2: Table S1. Combined effects of tooth loss and denture use on mortality [file 12903_2020_1084_MOESM2_ESM.docx]

**Additional Table 1.** Combined effects of tooth loss and denture use on mortality

|  | **Death** | **Participants** | **HR[95% CI]** |
| --- | --- | --- | --- |
| 0 tooth and no denture use | 2957 | 3661 | 1.00(reference) |
| 0 tooth and with denture use | 1808 | 3001 | 0.82[0.77, 0.87] |
| 1-9 teeth and no denture use | 3751 | 5147 | 0.91[0.87, 0.96] |
| 1-9 teeth and with denture use | 670 | 1238 | 0.75[0.69, 0.82] |
| 10-19 teeth and no denture use | 1619 | 2727 | 0.87[0.82, 0.93] |
| 10-19 teeth and with denture use | 262 | 628 | 0.61[0.53, 0.70] |
| 20+ teeth and no denture use | 1536 | 3849 | 0.75[0.70, 0.80] |
| 20+ teeth and with denture use | 169 | 565 | 0.60[0.51, 0.72] |

The effect was based on fully-adjusted model adjusting for age (years), sex (male or female), and residence (urban or rural), teeth number (0, 1–9, 10–19, ≥20, for analysis of denture use), denture use (yes or no, for analysis of teeth number), education (yes or no), sufficient income for daily needs (yes or no), co-residence (living alone or with others), BMI (<18.5, >=18.5 and <24, or >=24), smoking (current smoker, former smoker, or never smoker), drinking (current drinker, former drinker, or non- drinker), frequent vegetable consumption (yes or no), frequent fruit consumption (yes or no), impaired activity of daily living (yes or no), cognitive impairment(yes or no), hypertension (yes or no), self-reported history of diabetes mellitus (yes or no), self-reported history of heart disease (yes or no), self-reported history of cerebrovascular disease (yes or no), and self-reported history of respiratory diseases (yes or no).
