# Supplementary material for: Number of natural teeth, denture use and mortality in Chinese elderly: a population-based prospective cohort study
Source: BMC Oral Health. 2020 Apr 10;20:100. doi: 10.1186/s12903-020-01084-9 (PMC7147045; doi:10.1186/s12903-020-01084-9)
Supplement: Supplementary file 3 — Additional file 3: Table S2. Subgroup analysis for the associations between the number of natural teeth or denture use with mortality [file 12903_2020_1084_MOESM3_ESM.docx]

**Additional Table 2.** Subgroup analysis for the associations between the number of natural teeth or denture use with mortality

| **Subgroup** | **HR[95% CI] according to number of natural teeth** | | | | | |  | **HR[95% CI] according to denture use** | | | |
| --- | --- | --- | --- | --- | --- | --- | --- | --- | --- | --- | --- |
|  | **Death** | **Participant** | **20+** | **10-19** | **1-9** | **0** |  | **Death** | **Participant** | **No** | **Yes** |
| **Age, years** |  |  |  |  |  |  |  |  |  |  |  |
| >=65 and <75 | 1243 | 4743 | 1.00 | 1.65[1.37, 1.98] | 1.40[1.19, 1.64] | 1.22[1.05, 1.42] |  | 1243 | 4743 | 1.00 | 0.72[0.63, 0.83] |
| >=75 and <85 | 2467 | 4624 | 1.00 | 1.35[1.19, 1.54] | 1.17[1.04, 1.32] | 1.08[0.95, 1.24] |  | 2467 | 4624 | 1.00 | 0.70[0.63, 0.77] |
| >=85 and <95 | 4544 | 6127 | 1.00 | 1.37[1.22, 1.54] | 1.18[1.06, 1.33] | 1.14[1.00, 1.30] |  | 4544 | 6127 | 1.00 | 0.80[0.75, 0.87] |
| >=95 years | 4540 | 5322 | 1.00 | 1.29[1.10, 1.51] | 1.20[1.02, 1.41] | 1.04[0.86, 1.26] |  | 4540 | 5322 | 1.00 | 0.86[0.79, 0.94] |
| *P-interaction* |  |  |  | *<0.001* | *<0.001* | *0.002* |  |  |  |  | *<0.001* |
| **Sex** |  |  |  |  |  |  |  |  |  |  |  |
| Men | 5392 | 9111 | 1.00 | 1.15[1.04, 1.26] | 1.25[1.15, 1.37] | 1.40[1.27, 1.53] |  | 5392 | 9111 | 1.00 | 0.76[0.71, 0.82] |
| Women | 7402 | 11705 | 1.00 | 1.12[1.01, 1.25] | 1.19[1.08, 1.31] | 1.30[1.18, 1.43] |  | 7402 | 11705 | 1.00 | 0.84[0.79, 0.89] |
| *P-interaction* |  |  |  | *0.65* | *0.27* | *0.30* |  |  |  |  | *0.02* |
| **Residence** |  |  |  |  |  |  |  |  |  |  |  |
| Urban | 4816 | 8249 | 1.00 | 1.16[1.03, 1.30] | 1.24[1.11, 1.37] | 1.34[1.20, 1.49] |  | 4816 | 8249 | 1.00 | 0.81[0.75, 0.87] |
| Rural | 7978 | 12567 | 1.00 | 1.13[1.03, 1.24] | 1.21[1.12, 1.32] | 1.34[1.23, 1.46] |  | 7978 | 12567 | 1.00 | 0.81[0.76, 0.86] |
| *P-interaction* |  |  |  | *0.77* | *0.76* | *0.98* |  |  |  |  | *0.41* |
| **BMI, kg/m^2^** |  |  |  |  |  |  |  |  |  |  |  |
| <18.5 | 6310 | 8946 | 1.00 | 1.22[1.10, 1.35] | 1.30[1.17, 1.44] | 1.30[1.17, 1.44] |  | 6310 | 8946 | 1.00 | 0.83[0.77, 0.89] |
| >=18.5 and <24 | 5216 | 9060 | 1.00 | 1.19[1.07, 1.32] | 1.23[1.12, 1.35] | 1.23[1.12, 1.35] |  | 5216 | 9060 | 1.00 | 0.80[0.74, 0.85] |
| >=24 | 1268 | 2810 | 1.00 | 1.07[0.89, 1.30] | 1.16[0.97, 1.39] | 1.16[0.97, 1.39] |  | 1268 | 2810 | 1.00 | 0.76[0.66, 0.87] |
| *P-interaction* |  |  |  | *0.87* | *0.96* | *0.58* |  |  |  |  | *0.09* |
| **Smoking status** |  |  |  |  |  |  |  |  |  |  |  |
| Current | 2405 | 4205 | 1.00 | 1.18[1.08, 1.28] | 1.24[1.15, 1.33] | 1.35[1.25, 1.45] |  | 2405 | 4205 | 1.00 | 0.82[0.78, 0.86] |
| Not current | 10389 | 16611 | 1.00 | 1.05[0.91, 1.21] | 1.22[1.07, 1.39] | 1.41[1.23, 1.62] |  | 10389 | 16611 | 1.00 | 0.77[0.69, 0.85] |
| *P-interaction* |  |  |  | *0.07* | *0.34* | *0.68* |  |  |  |  | *0.65* |
| **Drinking status** |  |  |  |  |  |  |  |  |  |  |  |
| Current | 2610 | 4420 | 1.00 | 1.19[1.09, 1.29] | 1.22[1.14, 1.32] | 1.35[1.25, 1.46] |  | 2610 | 4420 | 1.00 | 0.82[0.78, 0.86] |
| Not current | 10184 | 136396 | 1.00 | 0.99[0.85, 1.14] | 1.23[1.08, 1.40] | 1.32[1.15, 1.52] |  | 10184 | 136396 | 1.00 | 0.74[0.67, 0.82] |
| *P-interaction* |  |  |  | *0.13* | *0.29* | *0.63* |  |  |  |  | *0.07* |
| **Denture use** |  |  |  |  |  |  |  |  |  |  |  |
| Yes | 2862 | 5,432 | 1.00 | 1.02[0.83, 1.26] | 1.22[1.02, 1.46] | 1.30[1.10, 1.54] |  | NA | NA | NA | NA |
| No | 9932 | 15,384 | 1.00 | 1.17[1.09, 1.27] | 1.25[1.17, 1.35] | 1.39[1.29, 1.50] |  | NA | NA | NA | NA |
| *P-interaction* |  |  |  | *0.21* | *0.89* | *0.89* |  |  |  |  |  |
| **Number of natural teeth** |  |  |  |  |  |  |  |  |  |  |  |
| 20+ | NA | NA | NA | NA | NA | NA |  | 5357 | 6,662 | 1.00 | 0.78[0.66, 0.92] |
| 10-19 | NA | NA | NA | NA | NA | NA |  | 4378 | 6,385 | 1.00 | 0.71[0.61, 0.82] |
| 1-9 | NA | NA | NA | NA | NA | NA |  | 1681 | 3,355 | 1.00 | 0.81[0.74, 0.89] |
| 0 | NA | NA | NA | NA | NA | NA |  | 1378 | 4,414 | 1.00 | 0.80[0.75, 0.85] |
| *P-interaction* |  |  |  |  |  |  |  |  |  |  | *0.17* |

HR: hazard ratio; CI: confidence interval; BMI: body mass index; NA: not applicable.

Subgroup analyses were performed with fully-adjusted model, adjusting for age (years), sex (male or female), and residence (urban or rural), teeth number (0, 1–9, 10–19, ≥20, for analysis of denture use), denture use (yes or no, for analysis of teeth number), education (yes or no), sufficient income for daily needs (yes or no), co-residence (live alone or with others), BMI (<18.5, >=18.5 and <24, or >=24), smoking (current smoker, former smoker, or never smoker), drinking (current drinker, former drinker, or non- drinker), frequent vegetable consumption (yes or no), frequent fruit consumption (yes or no), impaired activity of daily living (yes or no), cognitive impairment(yes or no), hypertension (yes or no), self-reported history of diabetes mellitus (yes or no), self-reported history of heart disease (yes or no), self-reported history of cerebrovascular disease (yes or no), and self-reported history of respiratory diseases (yes or no). A factor was removed from the cox-regression model when it was considered as a subgroup factor.

When took the number of natural teeth as an ordinal variable and tested the overall interaction effect for other factors, only age showed a interaction effect (P < 0.001). The p-values for the interaction with sex, residence, BMI, smoking status, drinking status, and denture use, were > 0.05.
