# Supplementary material for: Number of natural teeth, denture use and mortality in Chinese elderly: a population-based prospective cohort study
Source: BMC Oral Health. 2020 Apr 10;20:100. doi: 10.1186/s12903-020-01084-9 (PMC7147045; doi:10.1186/s12903-020-01084-9)
Supplement: Supplementary file 4 — Additional file 4: Table S3. Sensitivity analysis for the association of the number of natural teeth or denture use with mortality [file 12903_2020_1084_MOESM4_ESM.docx]

**Additional Table 3.** Sensitivity analysis for the association of the number of natural teeth or denture use with mortality

|  | **HR[95%CI]** | |  |  |  |  |
| --- | --- | --- | --- | --- | --- | --- |
|  | **Additionally adjusting for depressive tendencies** | | **Additionally adjusting for marital status** | **Additionally adjusting for time of recruitment** | **Using baseline number of teeth and denture use as covariate** | **Excluding patients with a history of diabetes mellitus, heart disease, cerebrovascular disease, or respiratory diseases** |
| **Deaths** | 12756 | | 12754 | 12757 | 12757 | 5273 |
| **Participants** | 20814 | | 20812 | 20816 | 20816 | 8816 |
| **Number of natural teeth** |  | |  |  |  |  |
| *20+* | 1.00 | | 1.00 | 1.00 | 1.00 | 1.00 |
| *10-19* | 1.14[1.06, 1.23] | | 1.13[1.05, 1.21] | 1.15[1.07, 1.23] | 1.18[1.10, 1.26] | 1.15[1.06, 1.25] |
| *1-9* | 1.23[1.15, 1.31] | | 1.20[1.13, 1.28] | 1.23[1.16, 1.32] | 1.19[1.12, 1.27] | 1.23[1.14, 1.32] |
| *0* | 1.35[1.26, 1.44] | | 1.32[1.24, 1.41] | 1.36[1.27, 1.45] | 1.24[1.16, 1.32] | 1.34[1.24, 1.45] |
| **Denture use** | |  | |  |  |  |
| *No* | 1.00 | | 1.00 | 1.00 | 1.00 | 1.00 |
| *Yes* | 0.81[0.77, 0.84] | | 0.81[0.78, 0.85] | 0.80[0.76, 0.84] | 0.88[ 0.84, 0.92] | 0.80[0.76, 0.84] |

HR: hazard ratio; CI: confidence interval;

Sensitivity analyses were based on fully-adjusted models, adjusting for age (years), sex (male or female), and residence (urban or rural), teeth number (0, 1–9, 10–19, ≥20, for analysis of denture use), denture use (yes or no, for analysis of teeth number), education (yes or no), sufficient income for daily needs (yes or no), co-residence (living alone or with others), BMI (<18.5, >=18.5 and <24, or >=24), smoking (current smoker, former smoker, or never smoker), drinking (current drinker, former drinker, or non- drinker), frequent vegetable consumption (yes or no), frequent fruit consumption (yes or no), impaired activity of daily living (yes or no), cognitive impairment(yes or no), hypertension (yes or no), self-reported history of diabetes mellitus (yes or no), self-reported history of heart disease (yes or no), self-reported history of cerebrovascular disease (yes or no), and self-reported history of respiratory diseases (yes or no).

**Additional Table 3** Sensitivity analysis for the association of the number of natural teeth or denture use with mortality **(continue)**

|  | **HR[95%CI]** | |  |  |  |  |  |
| --- | --- | --- | --- | --- | --- | --- | --- |
|  | **Excluding participants with an observation time of < 3 years** | | **Excluding participants with an observation time of > 12 years** | **Excluding participants without tooth loss** | **Considering the participants with unknown survival status censored at median of follow-up (3 years)** | **Additionally adjusting for location of living** | **Excluding the participants with increased number of teeth between two waves of survey** |
| **Deaths** | 6442 | | 12711 | 12319 | 12757 | 12757 | 10325 |
| **Participants** | 12,589 | | 19702 | 19570 | 20816 | 20816 | 12936 |
| **Number of natural teeth** |  | |  |  |  |  |  |
| *20+* | 1.00 | | 1.00 | 1.00 | 1.00 | 1.00 | 1.00 |
| *10-19* | 1.17[1.06, 1.30] | | 1.13[1.05,1.22] | 1.15[1.07, 1.24] | 1.15[1.09,1.22] | 1.14[1.06, 1.23] | 1.20[1.09, 1.37] |
| *1-9* | 1.32[1.21, 1.46] | | 1.22[1.15,1.30] | 1.23[1.15, 1.32] | 1.23[1.17,1.30] | 1.23[1.15,1.31] | 1.27[1.17, 1.37] |
| *0* | 1.35[1.26, 1.44] | | 1.34[1.25,1.43] | 1.35[1.26, 1.45] | 1.31[1.24,1.38] | 1.35[1.26, 1.44] | 1.23[1.13, 1.34] |
| **Denture use** | |  | |  |  |  |  |
| *No* | 1.00 | | 1.00 | 1.00 | 1.00 | 1.00 | 1.00 |
| *Yes* | 0.75[0.70, 0.79] | | 0.81[0.78,0.85] | 0.80[0.77, 0.84] | 0.85[0.82,0.88] | 0.81[0.77, 0.84] | 0.82[0.78, 0.86] |

HR: hazard ratio; CI: confidence interval;

Sensitivity analyses were based on fully-adjusted models, adjusting for age (years), sex (male or female), and residence (urban or rural), teeth number (0, 1–9, 10–19, ≥20, for analysis of denture use), denture use (yes or no, for analysis of teeth number), education (yes or no), sufficient income for daily needs (yes or no), co-residence (living alone or with others), BMI (<18.5, >=18.5 and <24, or >=24), smoking (current smoker, former smoker, or never smoker), drinking (current drinker, former drinker, or non- drinker), frequent vegetable consumption (yes or no), frequent fruit consumption (yes or no), impaired activity of daily living (yes or no), cognitive impairment(yes or no), hypertension (yes or no), self-reported history of diabetes mellitus (yes or no), self-reported history of heart disease (yes or no), self-reported history of cerebrovascular disease (yes or no), and self-reported history of respiratory diseases (yes or no).
